# Denture use and a slower rate of cognitive decline among older adults with partial tooth loss in China: A 10‐year prospective cohort study

**DOI:** 10.1002/agm2.12383

**Published:** 2024-12-23

**Authors:** Xiang Qi, Zheng Zhu, Yaolin Pei, Bei Wu

**Affiliations:** ^1^ Rory Meyers College of Nursing New York University New York New York USA; ^2^ School of Nursing Fudan University Shanghai China

**Keywords:** cognition, dementia, oral health

## Abstract

**Objective:**

Denture use has been shown to improve nutritional intake and diet quality in people with tooth loss. Despite evidence linking tooth loss and dementia, few studies have examined the association between denture use and cognitive decline. We investigated the relationship between denture use and cognitive decline among Chinese older adults with tooth loss.

**Methods:**

We analyzed data from the Chinese Longitudinal Healthy Longevity Survey 2008–2018, including 27,708 community‐dwelling dentate and edentulous (i.e., who have lost all natural teeth) older adults aged 65 and older. Cognitive function was assessed using the Mini‐Mental State Examination from 2008 to 2018. Linear mixed‐effect models were employed to assess the association of denture use with baseline cognitive function and rate of cognitive decline, adjusting for sociodemographic characteristics, health‐related behavior, and health status. Subgroup analyses evaluated differences in associations among dentate participants with varying degrees of tooth loss (1–9, 10–19, 20–31).

**Results:**

Compared to non‐denture users, dentate participants who used dentures had better baseline cognitive function (*β*, 1.032; 95% CI, 0.813–1.251; *p* < 0.001) and a slower annual decline in cognitive function (*β*, 0.127; 95% CI, 0.047–0.206; *p* < 0.01). For edentulous participants, denture use was associated with higher baseline cognitive function (*β*, 3.063; 95% CI, 2.703–3.423; *p* < 0.001) but not with the rate of cognitive decline (*β*, 0.011; 95% CI, –0.082 to 0.105; *p* = 0.818). Results remained consistent across subgroups of dentate participants with various degrees of missing teeth.

**Conclusions:**

Denture use may help protect against cognitive decline in older adults with partial tooth loss. This study highlights the potential importance of prosthodontic rehabilitation in preserving cognitive health. Further research is needed to establish a causal relationship between denture use and cognitive function.

## Graphical abstract

This 10‐year study of 27,708 Chinese older adults found denture use was associated with better baseline cognitive function and a slower rate of cognitive decline in participants with partial tooth loss. Findings suggest prosthodontic rehabilitation may help preserve cognitive health in aging.

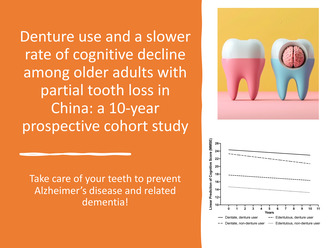

## 1 INTRODUCTION

Dementia, characterized by a progressive decline in cognitive functioning, can have devastating effects on individuals and their families.^1^ During the long preclinical phase of dementia, accelerated cognitive decline is considered one of the essential markers.^2^ Thus, it is important to identify modifiable factors that may prevent or delay the progression of cognitive decline.^3^, ^4^ Tooth loss can negatively impact chewing, speaking, and social engagement;^5^ all of which have been linked to cognitive health in later life.^6^, ^7^ Previous studies have established a connection between masticatory dysfunction, a direct consequence of losing natural teeth,^8^ and an increased risk of cognitive impairment.^9^, ^10^, ^11^ Emerging research indicates a significant relationship between mastication and activity in the cerebral cortex. For example, mastication is believed to enhance cerebral blood flow, thereby augmenting oxygen levels in the areas of the brain such as the prefrontal cortex and hippocampus.^12^, ^13^, ^14^, ^15^ Consequently, impaired mastication could lead to cognitive decline.

It is important to have prosthodontic rehabilitation to increase chewing function for people who lost their natural teeth. Wearing dentures, a form of prosthodontic rehabilitation, can aid in restoring both aesthetics and chewing function.^16^ Denture use has been associated with improved oral function, potentially leading to better nutritional status and overall quality of life.^17^ However, the relationship between denture use and cognitive decline is not well established, as most existing studies have relied solely on cross‐sectional data.^9^, ^10^, ^11^, ^18^ While the benefits of denture use in terms of improved masticatory function are recognized,^19^ there is a notable scarcity of comprehensive, well‐structured prospective studies investigating the impact of denture use on cognitive outcomes for older adults with tooth loss.^20^, ^21^ China has the highest number of dementia cases in the world, accounting for 25% of the total globally.^22^ Examining the association between denture use and cognitive decline among Chinese older adults with tooth loss could help address knowledge gaps in this field, and the findings would have policy and practical implications for global populations.

This prospective study aims to examine the association between denture use and cognitive health in Chinese older adults who have lost natural teeth. Using data from the Chinese Longitudinal Healthy Longevity Survey (CLHLS) 2008–2018, we tested our hypothesis that denture use is associated with better cognitive function and a slower rate of cognitive decline among Chinese older adults with tooth loss over a 10‐year period.

## 2 METHODS

### 2.1 Study population and sample

We used data from the CLHLS, which was designed to collect information on an array of socioeconomic status, behaviors, healthcare utilization, well‐being, and health outcomes among Chinese older adults aged 65 and older.^23^ The baseline survey was conducted in 1998, with follow‐up surveys with replacements for deceased older adults that were completed in 2000, 2002, 2005, 2008/09, 2011/12, 2014, and 2018 (the most recent wave).^23^ The details of the study design and data collection of CLHLS were described previously.^23^

**FIGURE 1:**
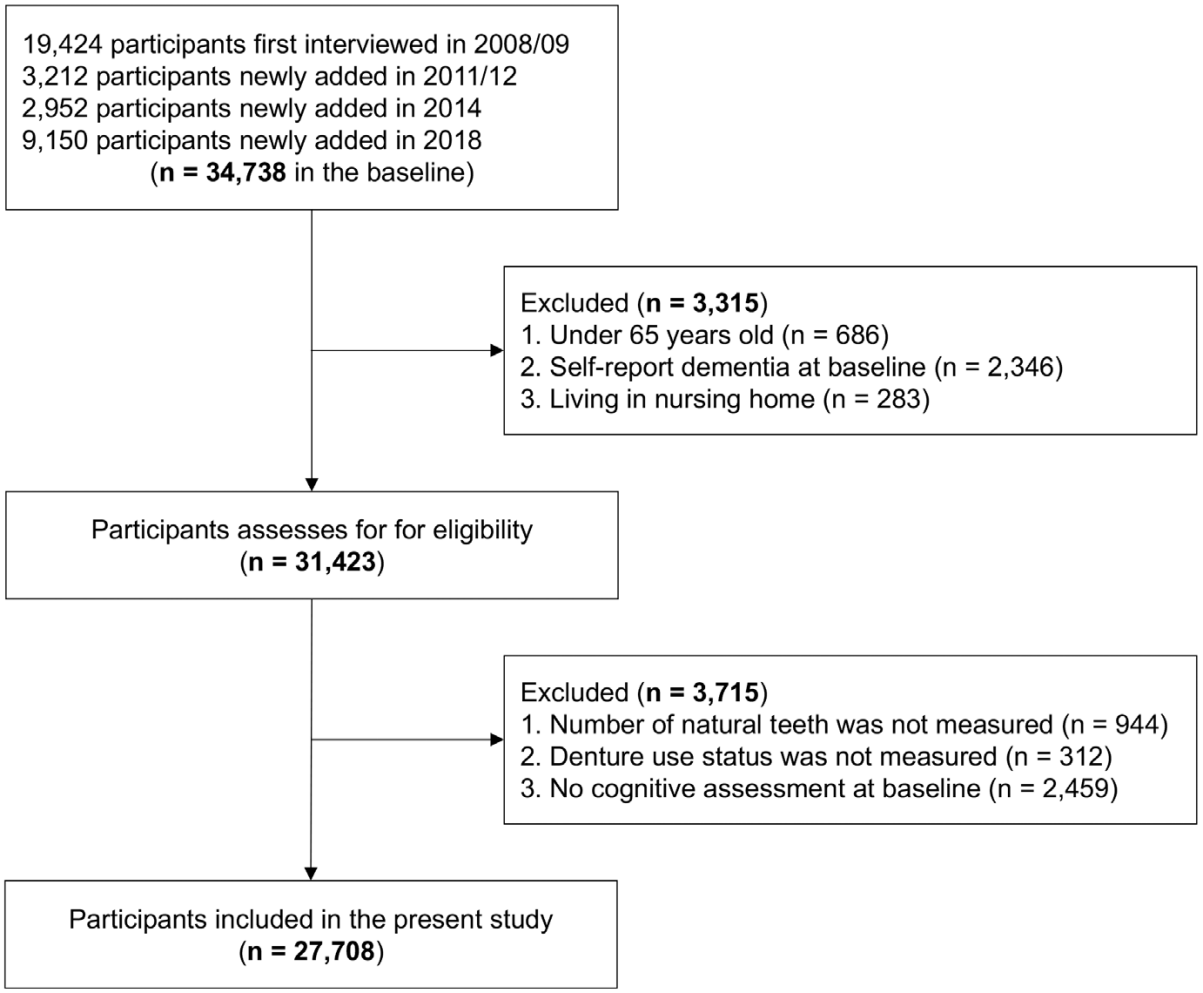
Flowchart for participant selection in this study.

The 2008/09 wave was the first time the CLHLS measured participants' dietary intake and diversity. Therefore, the latest four waves were selected for this study. Since the enrollment of CLHLS was continuous, new participants were recruited at each wave; thus, participants included in the study had different baselines and follow‐ups. The initial visit, during which cognitive function was assessed, served as the baseline. This study used data from the 2008/09 (*n* = 19,424), 2011/12 (*n* = 3212), 2014 (*n* = 2952), and 2018 (*n* = 9150) waves, where *n* refers to the number of newly added participants. A total of 7030 participants were excluded due to the following reasons: under 65 years old, with self‐reported dementia at the baseline, living in nursing homes, and no complete information on denture use, tooth loss, or cognitive assessments. No differences were found in baseline characteristics between participants included and those lost to follow‐up, except for depressive symptoms and diabetes. An analytical sample of 27,708 participants was included (Figure 1).

### 2.2 Measurements

#### 2.2.1 Denture use and tooth loss

Denture use and tooth loss were measured by two questions through in‐home interviews conducted by trained investigators: “Do you have dentures (referred to any type of non‐natural teeth, including partial or complete, removable, or implant‐retained fixed dentures)?” and “How many natural teeth do you still have (reported in numbers)?”. Because edentulism is the ultimate marker of oral morbidity and reflects people's history of oral disease, social circumstance, and use (or absence) of dental services,^24^ we divided the participants into two groups (dentate vs. edentulous) based on their number of lost teeth.

#### 2.2.2 Cognitive function

The Mini‐Mental State Examination (MMSE) was used to measure cognitive function.^25^ The MMSE captured six dimensions of older adults' cognitive ability: cognitive orientation, registration, attention, language, memory, and visuospatial ability.^26^ As previously documented,^25^ several items in the Chinese version of MMSE were modified to improve their meaning and cultural acceptability. The reliability and validity of the Chinese MMSE were demonstrated elsewhere.^25^ The total score of the Chinese MMSE is 30, with higher scores indicating better cognitive function.

#### 2.2.3 Covariates

Covariates were selected according to prior research.^27^, ^28^, ^29^ Participants' demographic characteristics included age, sex (male/female), and marital status (married vs. divorced/widowed/never married). Socioeconomic status was measured by education (years of schooling = 0 years, 1–6 years, >6 years), area of current residence (rural vs. urban), and financial sufficiency (financial support is sufficient to pay for daily expenses or not). Health‐related behaviors included smoking (never, ever, or current), drinking (never drink, stop drinking, or all the time), and regular consumption of fruit, vegetables, milk, and nuts (almost every day vs. occasionally/rarely/never).^28^ Health conditions included body mass index (BMI, underweight; normal; overweight),^30^ disability in activities of daily living (ADL, need help with at least one of their daily tasks: dressing, eating, toileting, bathing, indoor activities, and continence), depressive symptoms (yes/no), and self‐reported diagnosed hypertension and diabetes.^31^ The detailed definition of each covariate is shown in Table S1.

### 2.3 Statistical analysis

Participants' baseline characteristics were summarized according to denture use (yes or no). Data were presented as mean (standard deviation, SD) for continuous variables and percentages (%) for categorical variables and were compared using independent two‐sample *t*‐tests or Pearson *χ* ^2^ tests, as appropriate. All statistical analyses were conducted using Stata MP 17.0 (StataCorp LP, College Station, TX). Two‐sided *p* < 0.05 was considered statistically significant.

We used linear mixed‐effect models to examine the association of denture use with baseline cognitive function and the rate of cognitive decline (in SD per year), with the intercept and slope of follow‐up time fitted as random effects at the participant level. The follow‐up time was calculated by subtracting the date of the baseline cognitive function assessment from the dates of the cognitive function assessment in subsequent waves. Model 1 was an unadjusted model. Model 2 was adjusted for sociodemographic factors, including age, sex, living arrangement, marital status, education, residence, and financial sufficiency. Model 3 was constructed by adding health‐related behavior (smoking, drinking, and fruit/vegetable/milk/nut consumption) in Model 2. The final model (Model 4) was further adjusted for health status, including BMI, ADL disability, hypertension, and diabetes. Results were presented as *β* coefficients and 95% CIs. Variables with missing values for the total study are financial sufficiency (*n* = 231, 0.8%), drinking (*n* = 1071, 3.9%), and depressive symptoms (*n* = 936, 3.4%). We conducted a complete case analysis since missing data were rare.^32^

We further conducted subgroup analysis by the varying degrees of missing teeth to reflect the masticatory function of older adults with partial tooth loss. Guided by previous studies,^27^, ^28^ we categorized the number of missing teeth into three categories (1–9, 10–19, and, 20–31) and examined the effect of denture use on the three subgroups separately.

We conducted four sensitivity analyses. First, we additionally adjusted for the recruitment year to examine whether the cohort difference was associated with cognitive outcomes. Secondly, to assess attrition bias, participants who completed a cognitive function assessment at baseline were divided into two groups according to whether they had follow‐up visits. Third, although missing data were rare (<4%) in this study, we used an inverse probability weighting analysis to assess whether the missing data affected the results. Detailed descriptions are provided in the Supplementary Methods. Finally, we excluded participants whose denture use status changed during follow‐up to check the robustness of the results.

## 3 RESULTS

### 3.1 Baseline characteristics

We included 27,708 participants with a mean (SD) age of 86.0 (12.0), among whom 12,025 (43.4%) were men, 9747 (35.2%) were edentulous, and 8695 (31.4%) used dentures. The mean follow‐up time is 4.7 years (ranging from 0 to10 years). Participants without dentures were generally older, more likely to be women, less educated, either widowed or never married, residing in rural areas, and experiencing financial insufficiency (*p* < 0.05). Furthermore, this group had a higher likelihood of being current smokers and having a diet with lower intake of fruits, vegetables, milk, and nuts (*p* < 0.001). They also exhibited more ADL disability, more depressive symptoms, and were more prone to hypertension and diabetes compared to those with dentures (*p* < 0.001).

### 3.2 Denture use with cognitive health outcomes among dentate and edentulous participants

Table 2 presents the results from the linear mixed‐effect models. As shown in Model 1, denture use was significantly associated with a higher cognitive function at baseline (*β*, 3.091; 95% CI, 2.880–3.311; *p* < 0.001). After adjusting for potential covariates (Model 4), compared with non‐denture users, participants with dentures had higher cognitive function scores (*β*, 1.453; 95% CI, 1.273–1.632; *p* < 0.001) at baseline for the entire sample. For dentate participants (*n* = 17,961), denture users had an average 1.03‐point higher cognitive score at baseline (*β*, 1.032; 95% CI, 0.813–1.251; *p* < 0.001). For edentulous older adults (*n* = 9747), denture use was also associated with higher cognitive function at baseline (*β*, 3.063; 95% CI, 2.703–3.423; *p* < 0.001).

**FIGURE 2:**
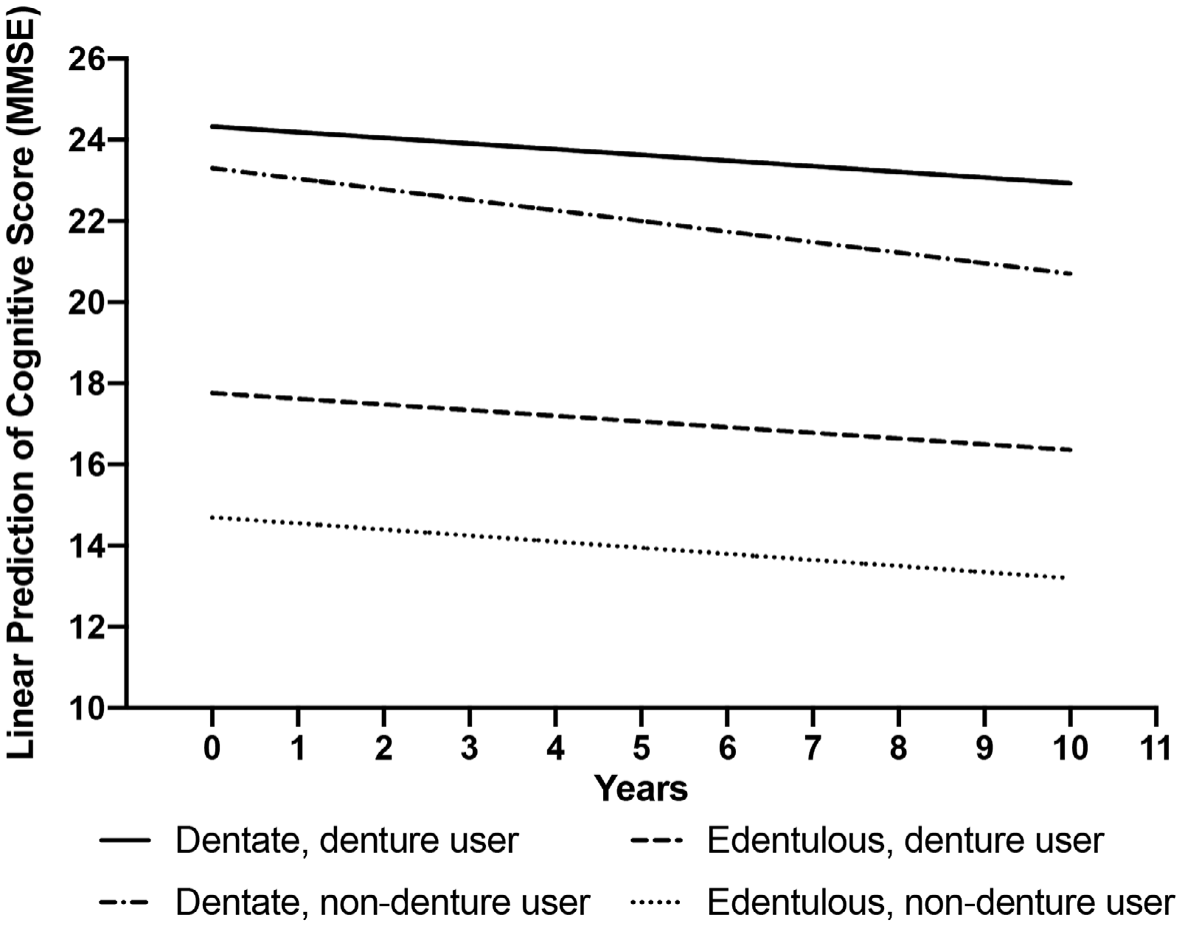
Estimated cognition function scores according to edentulism and denture status. Estimated cognitive function scores were calculated in SD units. The Model was controlled for age, sex, living arrangement, marital status, education, urban/rural residency, financial sufficiency, smoking, drinking, vegetable consumption, fruit consumption, nut consumption, milk consumption, body mass index, disability in activities of daily living, depressive symptoms, hypertension, and diabetes.

Figure 2 shows the estimated cognitive scores based on edentulism and denture status during follow‐ups. Results from fully adjusted models show that denture use was associated with on average 0.062 points less decline in cognitive score per year in the entire sample (*β*, 0.062; 95% CI, 0.022–0.101; *p* < 0.01; Table 2, Model 4). For dentate participants, denture use was significantly associated with, on average, 0.127 points less decline in cognitive score per year (*β*, 0.127; 95% CI, 0.047–0.206; *p* < 0.01). However, among edentulous participants, denture use was not associated with the rate of cognitive decline (*β*, 0.011; 95% CI, –0.082 to 0.105; *p* = 0.818).

### 3.3 Subgroup analysis by number of missing teeth among dentate participants

As shown in Table 3, the association of denture use with cognitive outcomes was generally consistent across dentate participants with different numbers of missing teeth. Specifically, for those with 20–31 missing teeth, there was a notable effect (*β*_intercept_, 1.392; 95% CI, 0.973–1.811; *β*_slope_, 0.132; 95% CI, 0.062–0.202), as well as in those with 10–19 missing teeth (*β*_intercept_, 0.582; 95% CI, 0.161–1.003; *β*_slope_, 0.107; 95% CI, 0.019–0.195), and those with 1–9 missing teeth (*β*_intercept_, 0.495; 95% CI, 0.185–0.805; *β*_slope_, 0.151; 95% CI, 0.072–0.230).

### 3.4 Sensitivity analysis

First, in sensitivity analyses that adjusted for the year of recruitment, no cohort differences were detected (Table S2). Second, compared with participants with two or more visits (*n* = 12,430), those who only had baseline visits (*n* = 15,278; a total of 9150 participants were newly added in 2018 and did not have follow‐ups) during the study period had lower levels of cognitive function, were less likely to use dentures, and had poorer health conditions. However, they did not differ in age, sex, education, drinking, and BMI (*p* > 0.05). By excluding participants with only baseline visits, the results were similar to the findings presented in the primary analyses (Table S3). Third, the inverse probability weighting analysis findings showed no meaningful differences from those of the primary analysis (Table S4). Finally, in another sensitivity analysis that excluded 2620 participants whose status of denture use changed in the follow‐up, the results were similar to those in the primary analyses (Table S5).

## 4 DISCUSSION

Using a nationally representative sample from the CLHLS 2008–2018, we found that denture use was associated with better cognitive function at baseline and a slower rate of cognitive decline over time among dentate participants. However, for edentulous participants, while denture use was linked to better baseline cognitive function, it was not significantly associated with the rate of cognitive decline. These findings remained consistent across various subgroup analyses and multiple sensitivity tests, suggesting that denture use may play a protective role against cognitive decline, particularly for those with partial tooth loss.

Our findings regarding the association between denture use and better cognitive health are consistent with prior studies. Cross‐sectional studies proposed that preserving more natural teeth and restoring lost teeth with dental prostheses, such as removable full or partial dentures, is related to better cognitive function.^21^, ^33^ Additionally, another cross‐sectional study highlighted that a diminished chewing function is linked to an increased risk of cognitive impairment, with this association being more pronounced in participants who did not use a partial denture or full denture.^11^ Furthermore, a four‐year longitudinal study from Japan observed that older adults with fewer teeth and no dentures faced a 1.85 times higher risk of dementia compared to those with 20 or more natural teeth.^34^ A meta‐analysis found that more missing teeth are associated with higher risks of cognitive impairment and dementia; however, the association was nonsignificant for participants using dentures.^35^ Collectively, these studies highlight the important role of good oral health in maintaining cognitive function and suggest that timely prosthodontic treatment could potentially slow down the progression of cognitive decline.

Various mechanisms have been proposed to elucidate the observed correlations between dentition or mastication and cognitive function. Previous research indicates that adults with suboptimal dentition (≤20 natural teeth) may exhibit lower nutritional intakes and poorer diet quality compared to those with full or moderate dentition (≥21 natural teeth),^36^ potentially leading to nutritional deficiencies and contributing to Alzheimer's disease development.^37^ In the current study, those with dentures tended to have more frequent fruit, vegetable, milk, and nut consumption than those without dentures (Table 1), which corresponds with a recent study that found older adults with poor dentition status (≤20 natural teeth) were associated with lower dietary diversity and worse nutritional status.^38^ Additionally, treatment with complete dentures or implant‐supported prostheses significantly improves nutritional status and oral health perception in older adults, thereby potentially enhancing cognitive function.^39^

**TABLE 1:** Characteristics of participants at baseline, and by denture use status, Chinese Longitudinal Healthy Longevity Survey, 2008–2018.

Variables	All participants (*n* = 27,708)	Dentate participants (*n* = 17,961)			Edentulous participants (*n* = 9747)		
		With denture (*n* = 3972)	No denture (*n* = 13,989)	*p‐*Value^a^	With denture (*n* = 4723)	No denture (*n* = 5024)	*p*‐Value^a^
Number of teeth, *M* ± SD	8.7 ± 10.1	13.8 ± 9.8	12.2 ± 8.7	<0.001	/		/
Cognitive function^b^, *M* ± SD	22.0 ± 9.4	26.5 ± 5.4	23.3 ± 8.5	<0.001	22.2 ± 9.0	14.7 ± 10.8	<0.001
Age, *M* ± SD	86.0 ± 12.0	79.5 ± 10.5	83.8 ± 11.9	<0.001	88.3 ± 10.4	95.4 ± 8.1	<0.001
Sex, %							
Men	43.4%	52.2%	46.3%	<0.001	43.2%	28.3%	<0.001
Women	56.6%	47.8%	53.7%		56.8%	71.7%	
Education, %							
0 year	57.7%	57.1%	81.7%	<0.001	36.2%	55.5%	<0.001
1–6 year (s)	29.0%	31.8%	14.9%		38.1%	30.5%	
≥6 years	13.3%	11.1%	3.4%		25.7%	14.0%	
Residence, %							
Urban	12.9%	18.7%	12.7%	<0.001	13.5%	8.1%	<0.001
Rural	87.1%	81.3%	87.3%		86.5%	91.9%	
Living alone, %	16.1%	16.5%	16.1%	0.461	16.8%	14.9%	0.011
Not married, %	62.9%	42.9%	58.2%	<0.001	67.4%	87.3%	<0.001
Financial insufficient, %	18.9%	14.3%	20.3%	<0.001	14.9%	22.4%	<0.001
Smoking, %							
Never	63.2%	62.2%	65.8%	0.011	64.4%	67.1%	<0.001
Ever	20.0%	18.2%	16.4%		19.0%	21.0%	
Current	16.8%	19.6%	17.8%		16.6%	11.9%	
Drinking, %							
Never	73.2%	71.5%	70.4%	0.251	70.9%	68.2%	<0.001
Stopped	10.3%	9.1%	12.0%		13.3%	20.0%	
All the time	16.5%	19.4%	17.6%		15.9%	11.8%	
Regular fruit consumption, %	41.4%	52.1%	39.4%	<0.001	49.1%	31.4%	<0.001
Regular vegetable consumption, %	87.9%	92.8%	88.9%	<0.001	88.5%	80.8%	<0.001
Regular milk consumption, %	32.9%	41.2%	28.5%	<0.001	42.1%	30.2%	<0.001
Regular nut consumption, %	13.4%	23.3%	12.3%	<0.001	17.3%	4.9%	<0.001
BMI (kg/m^2^), %							
Underweight, <18.5	26.5%	16.8%	25.1%	<0.001	26.0%	38.9%	<0.001
Normal, 18.5–23.9	52.5%	51.6%	53.8%		53.4%	48.4%	
Overweight, ≥24	21.0%	31.6%	21.1%		20.7%	12.7%	
Disability in ADL, %	22.9%	12.7%	17.3%	<0.001	28.0%	42.1%	<0.001
Depressive symptoms, %	34.7%	33.3%	32.1%	0.144	33.3%	44.5%	<0.001
Hypertension, %	27.6%	37.3%	27.6%	<0.001	28.8%	18.4%	<0.001
Diabetes, %	5.1%	9.4%	4.9%	<0.001	5.5%	1.6%	<0.001

Abbreviations: ADL, activities of daily living; BMI, body mass index calculated as weight in kilograms divided by the square of height in meters; *M* ± SD, mean ± standard deviation; MMSE, mini‐mental state examination.

^a^ Independent two‐sample *t*‐tests were used to compare the means of continuous variables. Pearson *χ* ^2^ tests were performed to compare the distribution of categorical variables.

^b^ Measured by mini‐mental state examination (scored 0–30), with a higher score indicating better cognitive function.

**TABLE 2:** Associations of denture use with baseline cognitive function and cognitive decline among dentate and edentulous older adults, Chinese Longitudinal Healthy Longevity Survey, 2008–2018.

	Model 1	Model 2	Model 3	Model 4
	*β* coefficient (95% confidence interval)			
Difference in baseline cognitive function between non‐denture users (reference) and denture users				
All participants (*n* = 27,708)	3.091 (2.880, 3.311)***	1.620 (1.441, 1.800)***	1.497 (1.315, 1.679)***	1.453 (1.273, 1.632)***
Dentate participants (*n* = 17,961)	3.195 (2.934, 3.446)***	1.224 (1.002, 1.446)***	1.111 (0.881, 1.330)***	1.032 (0.813, 1.251)***
Edentulous participants (*n* = 9747)	7.506 (7.136, 7.875)***	3.581 (3.231, 3.941)***	3.332 (2.970, 3.694)***	3.063 (2.703, 3.423)***
Difference in annual rate of change in cognitive function between non‐denture users (reference) and denture users				
All participants (*n* = 27,708)	−0.464 (–0.524, –0.403)***	0.121 (0.080, 0.162)**	0.101 (0.061, 0.142)**	0.062 (0.022, 0.101)**
Dentate participants (*n* = 17,961)	−0.315 (–0.441, –0.189)***	0.185 (0.121, 0.249)***	0.146 (0.084, 0.208)***	0.127 (0.047, 0.206)**
Edentulous participants (*n* = 9747)	−0.212 (–0.312, –0.133)***	−0.126 (–0.189, –0.064)***	0.031 (–0.061, 0.124)	0.011 (–0.082, 0.105)

*Note*: Model 1 was the crude model that was not adjusted for any covariates; Model 2 was adjusted for age, sex, living arrangement, marital status, urban/rural residency, education, and financial sufficiency. Model 3 was additionally adjusted for smoking, drinking, vegetable consumption, fruit consumption, nut consumption, and milk consumption; Model 4 was additionally adjusted for body mass index, disability in activities of daily living, depressive symptoms, hypertension, and diabetes.

*p < 0.05, **p < 0.01, ***p < 0.001.

**TABLE 3:** Subgroup analyses for the associations between denture use and cognitive decline among dentate older adults with different numbers of missing teeth.

Number of missing teeth	Difference in baseline cognitive function between non‐denture users (reference) and denture users	Difference in annual rate of change in cognitive function between non‐denture users (reference) and denture users
	*β* coefficient (95% confidence interval)	
20–31 (*n* = 7838)	1.392 (0.973, 1.811)***	0.132 (0.062, 0.202)*
10–19 (*n* = 4323)	0.582 (0.161, 1.003)**	0.107 (0.019, 0.195)*
1–9 (*n* = 5800)	0.495 (0.185, 0.805)**	0.151 (0.072, 0.230)***

*Note*: All models were adjusted for age, sex, living arrangement, marital status, education, urban/rural residency, financial sufficiency, smoking, drinking, vegetable consumption, fruit consumption, nut consumption, milk consumption, body mass index, disability in activities of daily living, depressive symptoms, hypertension, and diabetes.

*p < 0.05, **p < 0.01, ***p < 0.001.

An alternative hypothesis for the link between denture use and cognitive function involves increased cerebral blood flow due to jaw movements during mastication.^13^ Masticatory stimulation with normal occlusion has been shown to activate the brain, increase cerebral blood flow, and enhance oxygen levels in cortical areas.^15^ A study involving eight healthy individuals revealed that gum chewing was associated with heightened brain activation in the hippocampal and para‐hippocampal areas.^12^ Additionally, wearing partial dentures has been observed to increase activation in the dorsal prefrontal cortex and masseter muscle electromyography activity.^40^ Animal studies also demonstrated that mastication deficiency can lead to the degeneration of cholinergic neurons in the basal forebrain,^14^ hippocampal neuron loss, and suppression of learning ability,^41^ and consequently, impaired learning and memory in aged rats.^14^, ^41^ Another explanation is that the positive psychosocial impacts of wearing dentures and restoring aesthetics will lead to increased social engagement,^42^ which was well‐recognized to be associated with a lower risk of dementia in late life.^6^, ^43^

Notably, we found that accelerated cognitive decline still exists in edentulous participants with dentures. One explanation for the insignificant association is that the masticatory efficiency of denture wearers is not enough to match that of participants with full dentition. Consequently, dentures may not fully compensate for the loss of all natural teeth.^44^ Additionally, older adults with edentulism may experience a drastic change in diet, which could cause nutrient deficiency.^45^, ^46^ The average age of the participants in our study was 86.0 years; however, those edentulous individuals not using dentures had a higher average age of 95.4 years. This age difference suggests that those who are older may be more prone to cognitive decline, regardless of denture use, when compared to their younger counterparts.

Several limitations should be considered. First, CLHLS is a national health dataset that lacked detailed dental examination and treatment information, such as periodontal condition and masticatory function, potentially affecting the study results; however, adjustments for factors like dietary intake and health behaviors were made to reduce confounding. The absence of data on denture quality also limits assessment of the impact of denture use sites, duration, and causes. Second, the reliance on interview questionnaires for dental status, health behaviors, and health status could lead to recall bias. Third, a high attrition rate in follow‐ups may lead to survivorship bias. However, the inverse probability weighting analysis results were not materially changed. Finally, observational studies usually have some unknown residual confounding factors; therefore, the results reveal an association between the factors, not a causal relationship. Randomized clinical trials are needed to verify our findings.

## 5 CONCLUSION

The findings of our study indicate that denture use is associated with better baseline cognitive function and a slower rate of cognitive decline among Chinese older adults with partial tooth loss. These results add further evidence to the interrelationship between oral health and brain aging and highlight the need for research into the impact of prosthodontic rehabilitation on cognitive health. These findings have important clinical and public health implications. For example, it highlighted the need for more comprehensive universal dental care coverage, especially for older adults, among whom severe tooth loss and/or masticatory dysfunction are highly prevalent. This could also be a potential way to reduce dental rehabilitation access inequalities and minimize the negative effects of masticatory dysfunction on cognitive health. Although the results from this study suggest denture use may serve to counteract the detrimental effects of masticatory dysfunction on cognitive decline, more research is needed to evaluate the effectiveness of denture use on cognition outcomes further due to the considerable public health implications.

## AUTHOR CONTRIBUTIONS

XQ contributed to the conceptualization and design of the study, analyses, and interpretation of the results, and drafting the manuscript. ZZ contributed to the conceptualization of the study, interpretation of the results, and drafting of the manuscript. YP contributed to the conceptualization of the study, interpretation of the results, and drafting the manuscript. BW contributed to the conceptualization and design of the study, the interpretation of the results, and drafting the manuscript. All authors approved the final version to be published.

## FUNDING INFORMATION

This study is partially supported by National Institutes of Health (NIH)/National Institute of Aging (NIA) R01AG089856, P30AG083257, and K99AG076871; NIH/National Institute of Dental and Craniofacial Research (NIDCR) U01DE027512; and NIH/National Institute of Minority Health and Health Disparities (NIMHD) P50MD017356. The content is solely the responsibility of the authors and does not necessarily represent the official views of the NIH.

## CONFLICT OF INTEREST STATEMENT

The corresponding author (Dr. Bei Wu, New York University) is a member of *Aging Medicine*'s editorial board. We disclose this in accordance with the journal's conflict of interest policy. The authors declare no other potential conflicts of interest relevant to this manuscript.

## ETHICS STATEMENT

The Chinese Longitudinal Healthy Longevity Survey (CLHLS) study was approved by research ethics committees of Duke University and Peking University (IRB00001052‐13074). All participants provided written informed consent. This project is a secondary analysis of the CLHLS, which uses completely de‐identified public datasets. As this study did not involve direct human participants and utilized only publicly available data, per the Common Rule (45 CFR §46), institutional review board review and approval was not required.

## Supporting information


Appendix S1

## Data Availability

The CLHLS datasets are publicly available at the National Archive of Computerized Data on Aging (ICPSR 36179). Researchers may obtain the datasets after sending a data user agreement to the CLHLS team. http://www.icpsr.umich.edu/icpsrweb/NACDA/studies/36179.
