# Supplementary material for: Denture use and a slower rate of cognitive decline among older adults with partial tooth loss in China: A 10‐year prospective cohort study
Source: Aging Med (Milton). 2024 Dec 23;7(6):781–9. doi: 10.1002/agm2.12383 (PMC11702451; doi:10.1002/agm2.12383)
Supplement: Supplementary file 1 — Appendix S1 [file AGM2-7-781-s001.docx]

**Supplement Table S1. Measurement of study covariates.**

| **Variables** | **Type** | **Measurement** |
| --- | --- | --- |
| **Demographics** |  |  |
| Age | Continuous | In years |
| Sex | Binary | 1 = female; 2 = male |
| Living arrangement | Binary | 1 = living alone; 2 = living with others |
| Marital status | Binary | 1 = married; 2 = divorced/widowed/never married |
| **Socioeconomic status** |  |  |
| Education | Categorical | 0 = 0 year of schooling, 1-6 school years of schooling, >6 school years of schooling |
| Area of current residence | Binary | 1 = rural; 2= urban |
| Financial sufficiency | Binary | 1 = all source of financial support is sufficient to pay for daily expenses; 2 = not sufficient |
| **Health-related behavior** |  |  |
| Smoking | Categorical | 0 = never, 1 = ever, 2 = current |
| Drinking | Categorical | 0 = never drink, 1 = stop drinking; 2 = all the time |
| Regular consumption of fruit | Binary | 1 = almost every day; 2 = occasionally/rarely/never |
| Regular consumption of vegetable | Binary | 1 = almost every day; 2 = occasionally/rarely/never |
| Regular consumption of milk | Binary | 1 = almost every day; 2 = occasionally/rarely/never |
| Regular consumption of nut | Binary | 1 = almost every day; 2 = occasionally/rarely/never |
| **Health status** |  |  |
| Body mass index (BMI) | Categorical | 1 = underweight (BMI <18.5 kg/m^2^); 2 = normal (BMI 18.5–23.9 kg/m^2^); 3 = overweight (BMI ≥24 kg/m^2^) (Deng et al., 2020) |
| Disability in activities of daily living | Binary | A scale that contained six daily tasks: dressing, eating, toileting, bathing, indoor activities, and continence. When participants indicated that they were fully independent in performing all tasks, we coded this as 0. If they need help on any of these tasks, we code this variable as 1. |
| Depressive symptoms | Binary | Assessed by asking (a) have you felt fearful or anxious? (b) have you felt lonely and isolated? (c) have you felt useless with age? Participants who had answered “always” or “often” in all three questions were defined as having depressive symptoms. 1 = yes, 2 = no (Lyu & Sun, 2020). |
| Hypertension | Binary | 1 = self-report being diagnosed with hypertension by doctors; 2 = no |
| Diabetes | Binary | 1 = self-report being diagnosed with diabetes by doctors; 2 = no |

**Supplement Methods.**

Based on previous studies (Li et al., 2023; Seaman & White, 2013), weights were derived from a logistic regression analysis between a set of measures that were independently predictive of missing data (i.e., financial sufficiency, drinking, depressive symptoms, hypertension, and diabetes). We predict the probability that each individual is a complete case (i.e., non-missing) using logistic regression. The inverse of the above probabilities is the weight. Minimal missing data on indicators used to derive weights were singly imputed as the modal value of continuous variables or mean value of categorical variables (all indicators had <4% of values missing). The Hosmer-Lemeshow test was used assess the fit of the missingness model, with results showing no indication of poor fit (*P*-value < .05) (Nattino et al., 2020). Weights ranged from 2.21 to 4.61. The inverse probability weighting analysis is performed by adding weights in the primary analyses

**Supplementary Table S2. Sensitivity analysis for the associations of denture use with cognitive decline by adjusting for the recruitment year (*n* = 27,708).**

| **Groups** | **Difference in baseline cognitive function between non-denture users (reference) and denture users** | **Difference in annual rate of change in cognitive function between non-denture users (reference) and denture users** |
| --- | --- | --- |
|  | **β coefficient (95% confidence interval)** | |
| All participants (*n* = 27,708) | 1.346 (1.163,1.529)^***^ | 0.061 (0.020,0.102)^**^ |
| Dentate participants (*n* = 17,961) | 1.001 (0.761,1.242)^***^ | 0.125 (0.045,0.205)^**^ |
| Edentulous participants (*n* = 9,747) | 2.063 (1.702,2.424)^***^ | 0.020 (-0.071,0.110) |

Note: All models were adjusted for year of recruitment (2008/09, 2011/12, 2014, and 2018), age, sex, living arrangement, marital status, education, urban/rural residency, financial sufficiency, smoking, drinking, vegetable consumption, fruit consumption, nut consumption, milk consumption, body mass index, disability in activities of daily living, depressive symptoms, hypertension, and diabetes.

^*^ *P* < 0.05, ^**^ *P* < 0.01, ^***^ *P* < 0.001

**Supplementary Table S3. Sensitivity analysis for the associations of denture use with cognitive decline by excluding 15,278 participants with only baseline visit (*n* = 12,430).**

| **Groups** | **Difference in baseline cognitive function between non-denture users (reference) and denture users** | **Difference in annual rate of change in cognitive function between non-denture users (reference) and denture users** |
| --- | --- | --- |
|  | **β coefficient (95% confidence interval)** | |
| All participants (*n* = 12,430) | 0.951 (0.750,1.152)^***^ | 0.071 (0.030,0.112)^***^ |
| Dentate participants (*n* = 8,660) | 0.764 (0.521,1.007)^***^ | 0.065 (0.033,0.097)^***^ |
| Edentulous participants (*n* = 3,770) | 2.364 (1.562,3.166)^***^ | 0.005 (-0.201,0.211) |

Note: All models were adjusted for age, sex, living arrangement, marital status, education, urban/rural residency, financial sufficiency, smoking, drinking, vegetable consumption, fruit consumption, nut consumption, milk consumption, body mass index, disability in activities of daily living, depressive symptoms, hypertension, and diabetes.

^*^ *P* < 0.05, ^**^ *P* < 0.01, ^***^ *P* < 0.001

**Supplementary Table S4. The inverse probability weighting analysis: associations of denture use with cognitive decline (*n* = 27,708).**

| **Groups** | **Difference in baseline cognitive function between non-denture users (reference) and denture users** | **Difference in annual rate of change in cognitive function between non-denture users (reference) and denture users** |
| --- | --- | --- |
|  | **β coefficient (95% confidence interval)** | |
| All participants (*n* = 27,708) | 1.336 (1.124,1.548)^***^ | 0.105 (0.045,0.155)^***^ |
| Dentate participants (*n* = 17,961) | 0.931 (0.811,1.051)^***^ | 0.083 (0.031,0.135)^**^ |
| Edentulous participants (*n* = 9,747) | 2.242 (1.571,2.913)^***^ | 0.146 (-0.184,0.458) |

Note: Weights ranged from 2.21 to 4.61. The inverse probability weighting analysis is performed by adding weights in the primary analyses. All models were adjusted for age, sex, living arrangement, marital status, education, urban/rural residency, financial sufficiency, smoking, drinking, vegetable consumption, fruit consumption, nut consumption, milk consumption, body mass index, disability in activities of daily living, depressive symptoms, hypertension, and diabetes.

^*^ *P* < 0.05, ^**^ *P* < 0.01, ^***^ *P* < 0.001

**Supplementary Table S5. Sensitivity analysis for the associations of denture use with cognitive decline by excluding 2,620 participants whose status of denture use changed in the follow-up (*n* = 25,088).**

| **Groups** | **Difference in baseline cognitive function between non-denture users (reference) and denture users** | **Difference in annual rate of change in cognitive function between non-denture users (reference) and denture users** |
| --- | --- | --- |
|  | **β coefficient (95% confidence interval)** | |
| All participants (*n* = 25,088) | 1.431 (1.251,1.611)^***^ | 0.124 (0.048,0.200)^**^ |
| Dentate participants (*n* = 15,987) | 1.635 (1.319,1.951)^***^ | 0.164 (0.082,0.246)^***^ |
| Edentulous participants (*n* = 9,101) | 1.304 (1.064,1.544)^***^ | 0.001 (-0.120,0.121) |

Note: All models were adjusted for age, sex, living arrangement, marital status, education, urban/rural residency, financial sufficiency, smoking, drinking, vegetable consumption, fruit consumption, nut consumption, milk consumption, body mass index, disability in activities of daily living, depressive symptoms, hypertension, and diabetes.

^*^ *P* < 0.05, ^**^ *P* < 0.01, ^***^ *P* < 0.001

**References:**

Deng, Y., Gao, Q., Yang, D., Hua, H., Wang, N., Ou, F., Liu, R., Wu, B., & Liu, Y. (2020). Association between biomass fuel use and risk of hypertension among Chinese older people: A cohort study. *Environment International*, *138*, 105620. https://doi.org/10.1016/j.envint.2020.105620

Li, Y., Wang, W., Zhu, L., Yang, L., Wu, H., Zhang, X., Guo, L., & Lu, C. (2023). Pet Ownership, Living Alone, and Cognitive Decline Among Adults 50 Years and Older. *JAMA Network Open*, *6*(12), e2349241. https://doi.org/10.1001/jamanetworkopen.2023.49241

Lyu, S., & Sun, J. (2020). How does personal relative deprivation affect mental health among the older adults in China? Evidence from panel data analysis. *Journal of Affective Disorders*, *277*, 612–619. https://doi.org/10.1016/j.jad.2020.08.084

Nattino, G., Pennell, M. L., & Lemeshow, S. (2020). Assessing the goodness of fit of logistic regression models in large samples: A modification of the Hosmer‐Lemeshow test. *Biometrics*, *76*(2), 549–560. https://doi.org/10.1111/biom.13249

Seaman, S. R., & White, I. R. (2013). Review of inverse probability weighting for dealing with missing data. *Statistical Methods in Medical Research*, *22*(3), 278–295. https://doi.org/10.1177/0962280210395740
